# Early life adiposity and telomere length across the life course: a systematic review and meta-analysis

**DOI:** 10.12688/wellcomeopenres.13083.2

**Published:** 2018-08-07

**Authors:** Anna L. Guyatt, Santiago Rodriguez, Tom R. Gaunt, Abigail Fraser, Emma L. Anderson

**Affiliations:** 1 MRC Integrative Epidemiology Unit, Population Health Sciences, University of Bristol, Bristol, UK; 2 Population Health Sciences, University of Bristol, Bristol, UK; 3 NIHR Biomedical Research Centre at the University Hospitals Bristol NHS Foundation Trust, University of Bristol, Bristol, UK

**Keywords:** adiposity, telomere length, systematic review, children

## Abstract

**Background**: The relationship between adiposity at birth and in childhood, and telomere length is yet to be determined. We aimed to systematically review and meta-analyse the results of studies assessing associations between neonatal and later childhood adiposity, and telomere length.

**Methods**: We searched Medline, EMBASE and PubMed for studies reporting associations between adiposity measured in the neonatal period or later childhood/adolescence, and leucocyte telomere length, measured at any age via quantitative polymerase chain reaction, or terminal restriction fragment analysis, either cross-sectionally, or longitudinally. Papers published before April 2017 were included.

**Results**: Out of 230 abstracts assessed, 23 papers (32 estimates) were retained, from which 19 estimates were meta-analysed (15 cross-sectional, four longitudinal). Of the 15 cross-sectional estimates, seven reported on neonates: four used binary exposures of small-for-gestational-age vs. appropriate-for-gestational age (or appropriate- and large-for-gestational age), and three studied birth weight continuously. Eight estimates reported on later childhood or adolescent measures; five estimates were from studies of binary exposures (overweight/obese vs. non-obese children), and three studies used continuous measures of body mass index. All four longitudinal estimates were of neonatal adiposity, with two estimates for small-for-gestational-age vs. appropriate-for-gestational age neonates, and two estimates of birth weight studied continuously, in relation to adult telomere (49-61 years). There was no strong evidence of an association between neonatal or later childhood/adolescent adiposity, and telomere length. However, between study heterogeneity was high, and there were few combinable studies.

**Conclusions**: Our systematic review and meta-analysis found no strong evidence of an association between neonatal or later childhood or adolescent adiposity and telomere length.

## Introduction

Telomeres are regions of repetitive (TTAGGG) _n_ sequences situated at the ends of chromosomes. They buffer against loss of coding DNA (the ‘end replication problem’), and there is evidence that telomere length is associated with chronological age ^1^ and longitudinally with diseases of later life, such as cardiovascular disease ^2, 3^ and cancer ^3, 4^.

In addition to disease states, an association has been observed between unhealthy lifestyle factors and a reduction in telomere length ^5^. This has led to the suggestion that telomere length may lie on the causal pathway between traditional risk factors and chronic disease ^6^. One such studied risk factor is adiposity; there is evidence that greater adiposity in adults is associated with shorter telomere length, in both cross-sectional and longitudinal studies ^7, 8^. Given that obesity may result in chronic levels of inflammation and oxidative stress ^9^, and that telomeric DNA is vulnerable to damage by oxidative stress ^10^, it is plausible that obesity may promote telomere attrition ^7^.

Findings from existing studies that have assessed the association between obesity and leucocyte telomere length in children are conflicting, with studies reporting positive ^11^, negative ^12^ and null ^13– 19^ findings. Two systematic reviews of adiposity and telomere length that primarily focused on adiposity measured in adults have also briefly reported on evidence from studies of adiposity in childhood: Mundstock *et al.* ^8^ systematically reviewed and meta-analysed the results of three cross-sectional studies ^12– 14^ of the association between childhood obesity and telomere length ^8^. This review reported greater childhood adiposity to be associated with shorter telomere length. Müezzinler *et al.* ^7^ retrieved three studies assessing the association between body mass index (BMI) and telomere length in children, but concluded that none of the studies were suitable for meta-analysis ^7^. Additional studies have been published since these reviews. Furthermore, neither study assessed the association of adiposity at birth (as opposed to in later childhood) with telomere length. This is of interest for two reasons: firstly, in utero adversity is a predictor of later chronic diseases ^20^, for which telomere length may be a risk factor ^2, 3^, and secondly, telomere length is a marker of numerous adverse conditions across the life course, yet few studies have examined markers of prenatal adversity (a time of active cell replication) in relation to telomere length ^21^. Identifying associations in children (as opposed to adults) may also provide useful information about the ages at which associations between adiposity and telomere length emerge, and whether or not the direction and magnitude of the association between adiposity and telomere length is consistent through infancy, later childhood/adolescence and adulthood.

Here, we report the results of a systematic review and meta-analysis of both cross-sectional and longitudinal studies from the general population (i.e. in non-clinical populations) that have assessed the relationship between measures of neonatal and/or adiposity in older children and telomere length.

## Methods

### Inclusion criteria

Eligible studies included those with at least one measure of adiposity in the neonatal period or later childhood/adolescence (hereafter used interchangeably with ‘childhood’, defined as after the neonatal period [0–28 days], with mean age <19 years). Any measure of adiposity was considered, including (but not restricted to) BMI, weight, waist circumference, waist-to-hip ratio, waist-to-height ratio, skinfold thickness, fat mass, ponderal index, and birth weight. The outcome considered was leucocyte telomere length measured in peripheral venous or cord blood, by either quantitative polymerase chain reaction (qPCR) or terminal restriction fragment analysis (TRF). Leucocyte telomere length is commonly considered as a proxy for ‘whole-body’ ageing and biochemical stress, as well as being a risk factor for disease in its own right ^22^. We considered both cross-sectional studies in which adiposity and telomere length were measured concurrently and longitudinal studies in which adiposity was measured in the neonatal period/childhood and telomere length was measured after a follow-up period, i.e. in either childhood or adulthood.

Studies were included even if adiposity measures were not the primary exposure (for example, studies in which adiposity measures were measured as covariates) provided that a relationship between adiposity and telomere length was assessed. Papers were only included if adiposity exposures were adjusted for age and sex, or if effect estimates were adjusted for (or stratified by) age and sex. These criteria were relaxed if the estimate was based on a sample in which participants’ ages varied by a range of no more than three years, if exposure groups were matched by age or sex, or if it was shown that age or sex was not associated with telomere length in the population of interest.

### Exclusion criteria

Studies examining the effect of an intervention were not included, unless a pre-intervention, cross-sectional estimate of the relationship was provided. Furthermore, studies were excluded if participants were selected into the study on the basis of comorbidities (e.g. sleep apnoea, maternal stress, prematurity). Articles were also excluded if no full text was available from the British Library.

### Search strategy

Medline and EMBASE were searched using the Ovid platform. PubMed was also searched. Searches were run until April, 2017. Search terms included thesaurus terms (MeSH/Emtree) for ‘telomere length’, ‘adiposity’, ‘obesity’, ‘weight’ and ‘birth weight’. In addition, thesaurus terms for infants and children were used. Appropriate synonyms were identified for all terms above and entered into the search as keyword searches in the title and abstract. The search strategy is detailed in Supplementary File 1.

Studies were considered eligible for screening regardless of language, provided that a translator could be sourced within the department where the review was performed. Reference lists of pertinent papers were searched in order to identify additional studies that may have been missed by the search strategy.

Only peer-reviewed sources of evidence (journal articles, doctoral theses) were included. If there was evidence of dual publication of a study population, the largest population was used (provided that this was available in full-text form). Conference abstracts were not included, but relevant abstracts were cross-referenced against the search results to ensure that any follow-up peer-reviewed sources resulting from the same data were included.

### Study screening and selection

One reviewer (AG) screened all titles and abstracts and excluded those that were clearly ineligible according to the criteria above. Decisions on remaining titles were made after discussion between two researchers (AG and ELA). Data were extracted from relevant full-text articles by two researchers (AG and ELA), using a standardised extraction form. Study authors were contacted to clarify ambiguous results. Any disagreement between the two researchers performing data extraction was resolved by discussion. Supplementary Figure 1 – Supplementary Figure 2 show flowcharts detailing the review and extraction process.

### Statistical analyses

To facilitate the pooling of results according to different transformations of both exposures and the outcomes (e.g. normalisation, z-scoring, log-transformation), all estimates were standardised for the meta-analyses. Plot digitiser software [http://arohatgi.info/WebPlotDigitizer] was used to extract data from studies presenting differences in means in the form of bar charts. For studies presenting estimates of average telomere length by adiposity exposure groups (for example, in small-for-gestational-age neonates compared to normal- and large-for-gestational-age neonates), effect sizes were expressed as the difference in telomere length (in SD units) between the two groups. For studies that analysed adiposity and telomere length as continuous variables, effect sizes were expressed as change in telomere length (in SD units) per 1-SD unit increase in the exposure variable. Formulae used for calculating standardised effect estimates and their standard errors are provided in Supplementary File 2.

Estimates and standard errors were meta-analysed in Stata MP Version 13 (StataCorp, TX) with the ‘metan’ command, using random-effects models. In addition to combining estimates of adiposity at different ages in separate meta-analyses, we also conducted different analyses for binary and continuous exposures. Moreover, cross-sectional and longitudinal studies were also meta-analysed separately, since longitudinal studies may provide information on whether an association between telomere length and birth weight tracks across the life course. Heterogeneity was estimated using the I ^2^ statistic, which represents the percentage of the total observed variability that is due to true differences in effect estimates between studies rather than chance variation ^23^. Harmonisation of data in preparation for meta-analysis was performed in R (see script in Supplementary File 3). The Stata ‘.do’ file for the meta-analysis is available in Supplementary File 4.

## Results

### Literature search

A total of 427 papers that were published until April 22, 2017 were obtained after searching Medline, EMBASE and PubMed ( Supplementary Figure 3). A total of 230 titles remained for assessment. Supplementary Figure 3 shows a PRISMA ^24^ flow diagram detailing the exclusion process of search results. A completed ‘MOOSE’ (Meta-analyses Of Observational Studies in Epidemiology) checklist is included in Supplementary Figure 4. Supplementary Table 1– Supplementary Table 4 give details of all studies assessed, and the reasons for which they were excluded. All titles that passed screening were English language papers. A total of 23 relevant studies (32 estimates) were identified after full-text screening.

### Summary of retrieved studies

***Estimates not included in meta-analysis.*** Thirteen estimates were not meta-analysed, either because they reported no estimate, or because the study design was not combinable with any other extracted estimate. The characteristics of these studies, along with the 13 reported effect estimates, are given in Table 1.

**Table 1.** Summary of studies included in narrative synthesis only (13 estimates).

ID	Adiposity	Exposure	Temporality	Notes	Adipose Group	N Adipose	Lean Group	N lean	Units X	Units Y	Country	DNA	Method	Age Baseline	Age Follow- Up	Age Adjustment	Percent Male	Sex Adjustment	Additional Covariates	Reason not in meta-analysis	Estimate
**Strohmaier 2015**	BWT	Group	Mixed	Twins	Adipose twin	775	Lean twin	775	.	TS	Various	Multiple sources	qPCR	Neonates	Age range: 0–80 yrs	Twins, plus TL age-adj.		Same-sex twins, TL sex-adj.	.	Twin design, could not combine	Difference TL (between heavier and lighter twin) in top 10% and Bottom 90% BWT values (BWT values=difference in BWT between heavier and lighter twin) [95%CI]: -0.10 * [-0.18 *, -0.02 *] p=0.0145 N=775 twin pairs
**Wojcicki 2016** **[Neonatal BMI]**	BWT	Group	Long.	LGA vs. AGA	≥95 ^th^ C.	20	<95 ^th^ C.	183	.	bp	Central America	peripheral	qPCR	Neonates	Mean (SD): 4.9 (0.5) yrs	Age-adj. Cs, child age at DNA sample	46.8	Sex-adj. Cs	Adj. for repeated telomere- length measures	Could not combine	Difference TL in obese vs. non-obese children [95%CI]: -306.8 [-597.7 *, -15.9 *] p=0.04 N=203
**Okuda 2002**	BWT	Cont.	XS						g	kb	USA	cord	TRF	Mean (SD): 38.6 (1.8) wks	.	Adj. gest. age	50.6	No assoc. with sex	.	No estimate	“There was no significant relation of TRF lengths from blood … with birthweight, gestational age, and birthweight adjusted for gestational age.” N~165
**Kajantie 2012** **[Twin BWT]**	BWT	Cont.	Long.						kg	z	Finland	peripheral	qPCR	Mean (SD): 36.8 (4.0) wks	Mean (SD): 27.5 (2.0) yrs	Covariate	54	Covariate	Pat. SES, zygosity	Twin design, could not combine	Change TL per unit BWT in twin pairs [95%CI]: -0.950 [-3.718, 1.819] p~0.16 * N=124 twin pairs
**Kajantie 2012** **[Twin PI]**	PI	Cont.	Long.						kg/m ^3^	z	Finland	peripheral	qPCR	Mean (SD): 36.8 (4.0) wks	Mean (SD): 27.5 (2.0) yrs	Covariate	54	Covariate	Pat. SES, zygosity	Twin design, could not combine	Change TL per unit PI in twin pairs [95%CI]: 0.076 [-0.202, 0.353] p~0.69 * N=124 twin pairs
**Kajantie 2012 [PI]**	PI	Cont.	Long.						kg	z	Finland	peripheral	qPCR	Mean (SD): 39.8 (1.9) wks	Mean (SD): 61.5 (2.9) yrs	Covariate	46.7	Covariate	Pat. SES	Could not combine	Change TL per unit PI [95%CI]: -0.001 [-0.021, 0.019] p~0.98 * N=1894
**Wojcicki 2016** **[Childhood WC]**	WC	Group	XS	Obese vs. Nonob	≥90th C.	33	<90thC.	163	.	bp	Central America	peripheral	qPCR	Age: ~4yrs	.	Age-adj. Cs, child’s age at DNA sample	46.8	Sex-adj. Cs	Adj. for repeated telomere- length measures	Could not combine	Difference TL in obese vs. non- obese children [95%CI]: -190.5 [-474.4 *, 93.4 *] p=0.19 N=196
**Buxton 2014** **[Men]**	BMI	Cont.	Long.						kg/m ^2^	log TS	Finland	peripheral	qPCR	Mean (SD): 5.81 (0.89) yrs	Age: 31 yrs	Birth cohort	100	Stratified	Mat. parity, childhood SES, smoking, adult SES, children, batch	Could not combine	% Change TL per unit BMI at adiposity rebound [95%CI]: 0.40 [-1.20, 2.02] p=0.625 N=1774
**Buxton 2014** **[Women]**	BMI	Cont.	Long.						kg/m ^2^	log TS	Finland	peripheral	qPCR	Mean (SD): 5.61 (0.95) yrs	Age: 31 yrs	Birth cohort	0	Stratified	Mat. parity, childhood SES, smoking, adult SES, children, batch, age menarche	Could not combine	% Change TL per unit BMI at adiposity rebound [95%CI]: 1.71 [0.26, 3.18] p=0.041 N=1794
**Masi 2012 [FM]**	Fat mass	Cont.	XS						SD of % FM	TS	UK	peripheral	qPCR	Mean (SD): 15.1 (0.6) yrs.	.	Covariate	55.3	Covariate	Ethnicity, town	Could not combine	Change TL per unit FM [95%CI]: -0.013 [-0.030, 0.004] p=0.12 N=1080
**Masi 2012 [SF]**	Sum of skinfolds	Cont.	XS						SDs of log mm	TS	UK	peripheral	qPCR	Mean (SD): 15.1 (0.6) yrs.	.	Covariate	55.3	Covariate	Ethnicity, town	Could not combine	Change TL per unit SF [95%CI]: -0.011 [-0.028, 0.006] p=0.21 N=1080
**Masi 2012 [WC]**	WC	Cont.	XS						SDs of log cm	TS	UK	peripheral	qPCR	Mean (SD): 15.1 (0.6) yrs	.	Covariate	55.3	Covariate	Ethnicity, town	Could not combine	Change TL per unit WC [95%CI]: -0.003 [-0.019, 0.012] p=0.66 N=1080
**Bethan-court 2014**	WCht	Cont..	Long.						cm	TS	Philip- pines	peripheral	qPCR	Mean (SD): 14.7 (0.3) yrs	Mean (SD): 21.7 (0.3) yrs	Covariate	52.4	Covariate	Ht, income, WC *sex age *sex	Could not combine	Change TL per unit WCht [SE]: -0.00059 [-0.00314 *, 0.00196 *] p=0.647 N=1681

Abbreviations: adj.=adjusted; cov=covariate(s); Nonob=nonobese; TS=telomere-single gene ratio; qPCR=quantitative polymerase chain reaction; yrs=years;TL=telomere length; BWT=birth weight; 95%CI=95% confidence interval; N=sample size; BMI=body mass index; bp=base pairs; C(s)=centile; SD=standard deviation; g=grams; kb=kilobases; TRF=terminal restriction fragment; assoc.=association with; gest.=gestation; wks=weeks; kg=kilograms; z(s)=Z-score(s); Pat./Mat.= paternal/maternal; SES=socioeconomic status; PI=ponderal index; m=metres; WC=waist circumference (adjusted for height); Strat=stratified; FM=fat mass; SF=sum of skinfolds; mm=millimetres; cm=centimetres; ht=height. *=estimated from data: 95%CI estimated using formula 95%CI=estimate± (1.96*standard error); standard errors estimated as necessary from published p values, assuming two-tailed tests, degrees of freedom~sample N. P values estimated from t distributions, assuming df~sample N.

Seven of the 13 estimates not included in meta-analysis were of childhood adiposity exposures (waist circumference ^16, 25, 26^, fat mass ^16^, sum of skinfolds ^16^, or BMI ^11^). Adiposity measures were recorded either in early childhood (mean age ~5 years) ^11, 25^, or in adolescence (mean age ~15 years) ^16, 26^, and were studied as continuous ^11, 16, 26^ or grouped ^25^ exposures. Telomere length was measured either cross-sectionally ^16, 25^, or after a follow-up period (mean age at telomere measurement in longitudinal studies: 22 ^26^ and 31 years ^11^). Generally, point estimates were negative, but confidence intervals were consistent with no association between measures of childhood adiposity and telomere length. One study reported a weak positive association between BMI at approximately 5 years and telomere length at 31 years, but only in women ^11^.

Six of the 13 estimates not included in the meta-analysis studied neonatal adiposity, either as continuous ponderal index ^21^, or as continuous ^21, 27^, or categorical birth weight ^25, 28^. Of the six estimates, three were from twin studies ^21, 28^. One estimate was cross sectional ^27^, and five measured telomere length after a degree of follow-up (age range at follow-up: 5–80 years) ^21, 25, 28^. In both cross-sectional and longitudinal studies, there was no discernible pattern of associations between neonatal adiposity and telomere length.

***Estimates included in meta-analysis.*** The 19 estimates (from 19 studies) that were retained for meta-analysis are described in Table 2. Of these, 15 were cross-sectional and 4 were longitudinal. Of the 15 cross-sectional estimates, 7 reported on neonatal adiposity: 4 used binary exposures of small- vs. appropriate-for-gestational age (or appropriate- and large-for-gestational age) ^29– 32^, and 3 studied birth weight continuously ^33– 35^. Eight papers studied childhood adiposity (age range 2–17 years), of which 5 estimates were from studies of overweight/obese vs. non-obese children ^12– 15, 25^, and 3 were studies of body mass index as a continuous measure ^16– 18^. Longitudinal studies assessed neonatal adiposity, and telomere length after a follow-up (range: ~23–69 years) ^21, 36– 38^: two studied small- versus appropriate-for-gestational age neonates ^36, 37^, and two studied birth weight as a continuous exposure ^21, 38^.

**Table 2.** Summary of studies included in meta-analysis (19 studies, 19 estimates).

ID	Adiposity	Exposure	Temporality	Notes	Adipose Group	N Adipose	Lean Group	N Lean	Units X	Units Y	Country	DNA	Method	Age Baseline	Age Follow- Up	Age Adjustment	Percent Male	Sex Adjustment	Additional Covariates	Analysis
**Akkad 2006**	BWT	Group	XS	AGA vs. SGA	>10th C	38	<=3rdC	34	.	kb	UK	cord	TRF	Mean (SD): 39.1 (1.6) wks	.	Age-adj. Cs	47.2	Sex-adj. Cs	.	Neonatal: AGA vs. SGA (Difference in TL [SD]) (cross- sectional)
**Davy 2009**	BWT	Group	XS	No FGR vs. FGR	Z=-1 to 1	8	<5thC b/wt; <10thC PI	8	.	kb	Philippines	cord	TRF	Gest. 40 wks	.	Age-adj. Cs	.	Sex-adj. Cs	.	Neonatal: AGA vs. SGA (Difference in TL [SD]) (cross- sectional)
**Tellechea** **2015**	BWT	Group	XS	AGA vs. SGA	>=10th C	57	<10th C	12	.	T/S	Argentina	cord	qPCR	Mean (SD): 38.4 (2.4) wks	.	Age-adj. Cs	.	Sex-adj. Cs	.	Neonatal: AGA vs. SGA (Difference in TL [SD]) (cross- sectional)
**De Zegher** **2016**	BWT	Group	XS	AGA vs. SGA	Z>=-1	76	Z<-2	27	.	Norm. T/S	Spain	cord	qPCR	Mean (SD): 38.4 (1.3) wks	.	Age-adj. Zs	.	Sex-adj. Zs	Mat. age, BMI, gest. weight gain and gest. age. No assoc. sex	Neonatal: AGA vs. SGA (Difference in TL [SD]) (cross- sectional)
**Drury 2015**	BWT	Cont.	XS	.	.	.	.	.	g	T/S	USA	cord	qPCR	Mean (SD): 38.9 (1.3) wks	.	No assoc. age	53	Covariate	Race, mat. age at conception, mat. educ., pat. age	Neonatal: Change in TL [SD] per 1-SD birth weight (cross-sectional)
**Entringer** **2013**	BWT	Cont.	XS	.	.	.	.	.	g	T/S	USA	cord	qPCR	Mean (SD): 38.8 (1.4) wks	.	Covariate	48	Covariate	Obstetric complications, preg. specific- stress.	Neonatal: Change in TL [SD] per 1-SD birth weight (cross-sectional)
**Wojcicki** **2015**	BWT	Cont.	XS	.	.	.	.	.	p/tile	bp	USA	cord	qPCR	Tertiles: 39&39.8 wks	.	Age-adj. Cs	44.4	Covariate	Mat. BMI, mat. Educ.	Neonatal: Change in TL [SD] per 1-SD birth weight (cross-sectional)
**Al-Attas** **2010**	BMI	Group	XS	Obese vs. Nonob.	See Cole 2008	52	Normal BMI	96	.	kb	KSA	peripheral	TRF	Mean (SD): 9.1 (2.4) yrs	.	Age-adj. Cs	46.6	Sex-adj. Cs	.	Childhood: Obese vs. non-obese (Difference in TL [SD]) (cross- sectional)
**Alegria-** **Torres** **2016**	BMI	Group	XS	Overwt/ Obese vs. Nonob.	>85th C	13	Not stated	85	.	T/S	Mexico	peripheral	qPCR	Age 6–12 yrs	.	Age-adj. Cs	43.9	Sex-adj. Cs	.	Childhood: Obese vs. non-obese (Difference in TL [SD]) (cross- sectional)
**Buxton** **2011**	BMI	Group	XS	Obese vs. Nonob.	>97thC	471	<90thC	322	.	log T/S	France	peripheral	qPCR	Mean (SD): 11.4 (2.8) yrs	.	Age-adj. Cs	48	Sex-adj. Cs	.	Childhood: Obese vs. non-obese (Difference in TL [SD]) (cross- sectional)
**Wojcicki** **2016**	BMI	Group	XS	Obese vs. Nonob.	>=95 ^th^ C	63	<95 ^th^ C	137	.	bp	USA	peripheral	qPCR	Approx 4yrs	Mean (SD): 4.9(0.5) yrs	Age-adj. Cs	46.8	Sex-adj. Cs	Adjusted for repeated telomere- length measurements within individuals	Childhood: Obese vs. non-obese (Difference in TL [SD]) (cross- sectional)
**Zannolli** **2008**	BMI	Group	XS	Obese vs. Nonob.	Z>2	12	Normal BMI	41	.	bp	Italy	peripheral	TRF	Mean (SD): 8.2 (3.5) yrs	.	Age-adj. Zs	.	Sex-adj. Zs	.	Childhood: Obese vs. non-obese (Difference in TL [SD]) (cross- sectional)
**Masi 2012**	BMI	Cont.	XS	.	.	.	.	.	SDs of log kg/m ^2^	T/S	UK	peripheral	qPCR	Mean (SD): 15.1 (0.6) yrs	.	Covariate	55.3	Covariate	Ethnicity, town.	Childhood: Change in TL [SD] per 1-SD BMI (cross- sectional)
**Milne 2015**	BMI	Cont.	XS	.	.	.	.	.	Z score	kb	Australia	peripheral	qPCR	Age range: 3–9 yrs	.	Covariate	50	Covariate	.	Childhood: Change in TL [SD] per 1-SD BMI (cross- sectional)
**Needham** **2012**	BMI	Cont.	XS	.	.	.	.	.	kg/m^2	T/S	USA	peripheral	qPCR	Mean (SD): 9.9 (1.6) yrs	.	Covariate	48	Covariate	Ethnicity	Childhood: Change in TL [SD] per 1-SD BMI (cross- sectional)
**Shalev** **2014**	BWT	Group	Long.	AGA vs. SGA	Not stated	965	Not stated	72	.	T/S	NZ	peripheral	qPCR	Neonates	Age: 38 yrs	Age-adj. Cs	52	Sex-adj. Cs	No assoc. sex	Neonatal: AGA vs. SGA (Difference in TL [SD]) (longitudinal)
**De Melo** **2017**	BWT	Group	Long.	AGA vs. SGA	>=10thC<90th C	62	<10thC	34	.	kb	Brazil	peripheral	TRF	Age range: 37–42 wks	Mean (SD): 23.8(0.73) yrs	Age-adj. Cs	0	All female	.	Neonatal: AGA vs. SGA (Difference in TL [SD]) (longitudinal)
**Kajantie** **2012**	BWT	Cont.	Long.	.	.	.	.	.	kg	z	Finland	peripheral	qPCR	Mean (SD): 39.8 (1.9) wks	Mean (SD): 61.5(2.9) yrs	Covariate	46.7	Covariate	Pat. SES	Neonatal: Change in TL [SD] per 1-SD birth weight (longitudinal)
**Pearce** **2012**	BWT	Cont.	Long.	.	.	.	.	.	Z score	log bp	UK	peripheral	qPCR	Median (IQR): 40 (40- 40) wks	Age range: 49–51 yrs	Age-adj. Zs	37.7	Sex-adj. Zs+cov.	.	Neonatal: Change in TL [SD] per 1-SD birth weight (longitudinal)

Abbreviations: BWT=birth weight; BMI=body mass index; SGA/AGA=small-/appropriate-for-gestational age; FGR=foetal growth restriction; Overwt=overweight; Nonob.=non-obese; Z(s)=Z-score(s); C(s)=centile(s); XS=cross-sectional; Long.=longitudinal; g=grams; p/tile=percentile; z=z-score; kg=kilograms; m=metres; SD=standard deviation; TRF=terminal restriction fragment; qPCR=quantitative polymerase chain reaction; Gest.=gestation; wks=weeks; yrs=years; T/S=telomere-single gene; kb=kilobases; bp=base pairs; adj.=adjusted; Pat.Mat.=paternal/maternal; educ.=education; preg.=pregnancy; circ.=circumference.

### Meta-analyses

**Figure 1.**
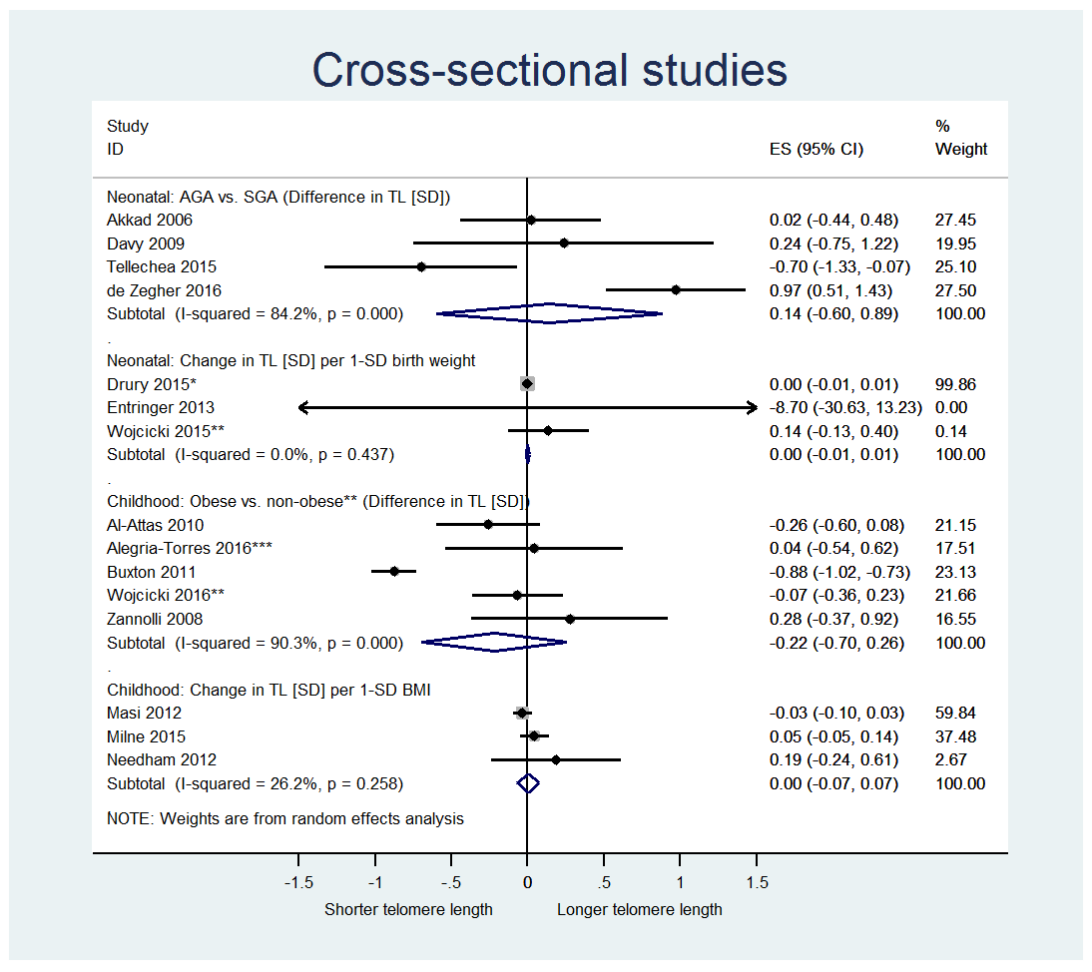
Meta-analyses of cross-sectional studies, separately by birth weight and BMI. Each panel shows a different sub-analysis, which is annotated in the ‘Study ID’ column. Meta-analysis is by random-effects, and 95% confidence intervals (CI) are shown (black horizontal bars), along with weights for each estimate. Box size is proportional to study weight, and black lines represent 95% CIs. Summary estimates for each panel are shown as diamonds. The null estimate is shown by the vertical black line. The scale is in standardised units (see Methods for more information). Specifically, subgroups labelled ‘Neonatal’ focus on studies of neonatal adiposity, and show the pooled estimates for the difference in telomere length (SD units) between small- and appropriate-for-gestational-age babies, and the change in telomere length (SD units), per 1-SD increase in birth weight. Subgroups labelled ‘Childhood’ examine the cross-sectional relationship between childhood or adolescent adiposity with telomere length, and show the difference in telomere length (SD units) for studies comparing groups of overweight/obese to non-overweight/obese children), and the change in telomere length (SD units) per 1-SD increase in BMI. Abbreviations: SGA/AGA=small-/appropriate-and-or-large-for-gestational age; TL=telomere length; SD=standard deviation; BMI=body mass index; ES=effect size. *=For Drury *et al*. (2015), the standard error was set to 0.00499, instead of 0.00, since a standard error of 0.00 prevented this estimate from being meta-analysed. Given that effect sizes, standard errors and confidence intervals were rounded to 2 decimal places in this paper, this approximates this largest value that this standard error could have taken, and still have been reasonably rounded to 0.00, as reported in the manuscript. **=Wojcicki *et al*. (2016) same population as Wojcicki *et al*. (2015) ***=Alegria-Torres *et al*. (2016) also included overweight children in the risk group (see Table 2). The names of the analyses in each panel correspond to those given in Table 2. P-values next to the I ^2^-value in each meta-analysis correspond to the p-value for the Q-statistics from the test of heterogeneity.

**Cross-sectional studies.** Figure 1 shows associations of cross-sectional studies of neonatal and childhood adiposity and telomere length. There was no evidence from these meta-analyses that neonatal adiposity or childhood adiposity were associated with concurrently measured telomere length.

**Figure 2.**
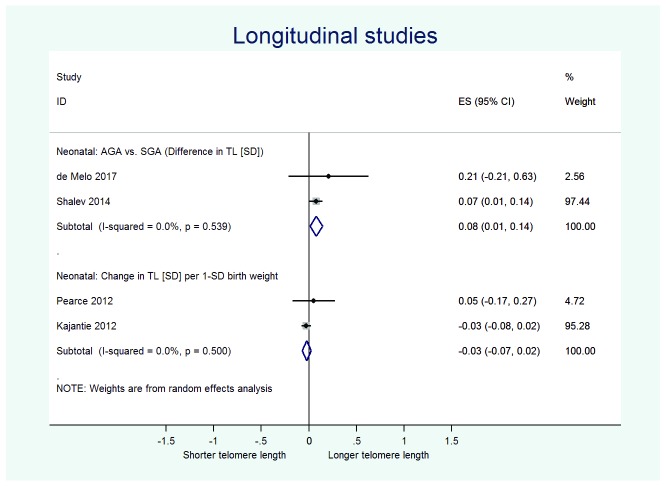
Meta-analyses of longitudinal studies, separately by birth weight and BMI. Each panel shows a different sub-analysis, which is annotated in the ‘Study ID’ column. Meta-analysis is by random-effects, and 95% confidence intervals (CI) are shown (black horizontal bars), along with weights for each estimate. Box size is proportional to study weight, and black lines represent 95% CIs. Summary estimates for each panel are shown as diamonds. The null estimate is shown by the vertical black line. This figure shows the difference in telomere length (SD units) for studies comparing telomere length in those born appropriate- and small-for-gestational-age, and the change in telomere length (SD units) per 1-SD increase in birth weight. Meta-analysis is by random-effects, and 95% confidence intervals (CI) are shown, along with weights for each estimate. Box size is proportional to study weight, and black lines represent 95% CIs. Summary estimates for each panel are shown as diamonds. The scale is in standardised units (see Methods for more information). Abbreviations: SGA/AGA=small-/appropriate-and-or-large-for-gestational age; TL=telomere length; SD=standard deviation; ES=effect size. The names of the analyses in each panel correspond to those given in Table 2. P-values next to the I ^2^-value in each meta-analysis correspond to the p-value for the Q-statistics from the test of heterogeneity.

**Longitudinal studies.** All longitudinal studies included in the meta-analysis measured adiposity only in neonates (i.e. no studies measured adiposity in childhood), with telomere length measured as early as 23.8 (SD 0.7) years ^37^ and as late as 69 years ^21^. Pooled estimates are shown in Figure 2. There was no evidence that continuously studied birth weight was associated with prospectively measured telomere length. There was very weak evidence that adults born appropriate-for-gestational age had longer telomeres than those born small-for-gestational age (SMD [95% CI]=0.08 [0.01-0.14]).

**Heterogeneity.** Heterogeneity in meta-analyses of non-continuous adiposity exposures was variable, but generally high (ranging from 0% to 90.3%). This suggests that as much as 90.3% of variation is due to true differences between studies and not due to chance. Heterogeneity was much lower in studies using continuous measures of adiposity (range: 0–26%).

## Discussion

We undertook a systematic review and meta-analysis of adiposity measured before 19 years of age in relation to longitudinal or cross-sectional estimates of telomere length measured in blood. To our knowledge, this is the first meta-analysis of adiposity and telomere length to synthesise evidence from neonatal measures of adiposity in relation to cross-sectionally or prospectively measured telomere length. We also provide updated estimates of the association of later childhood adiposity with telomere length ^7, 8^. We found no strong evidence for an association between any adiposity measure of neonatal or childhood adiposity and telomere length. A weak association suggesting that adults born small-for gestational age had shorter telomeres later in life was based on the meta-analysis of only two studies.

Generally, more heterogeneity was observed among effect estimates from studies assessing categorical adiposity measures (e.g. obese vs non-obese, small-for-gestational age vs. appropriate/large-for-gestational-age); I ^2^ estimates suggested that much of the between-study variation observed was due to true differences between studies and not due to chance. Conversely, very low heterogeneity was observed in the studies using continuous adiposity exposures. We were unable to formally assess possible sources of heterogeneity with meta-regression among studies using categorical adiposity measures, due to the small number of studies. However, heterogeneity is likely to be, at least in part, due to the differing thresholds used to define adiposity categories (e.g. percentiles of BMI), as well as other potential sources, such as differing ethnicities between studies, and the methods used to measure telomere length.

### Mechanisms for the association of adiposity and telomere length

It has been suggested that oxidative stress and inflammation are determinants of telomeric attrition, and it is proposed that as a source of oxidative stress ^9^ obesity may accelerate loss of telomeric DNA ^39^. When considered as a non-causal biomarker of ageing, the shortening of telomere length as a result of inflammation and oxidative stress is known as the ‘telomeric clock’ model ^40^. However, there is evidence that there is a complex ‘axis of ageing’ that exists between telomeres and mitochondrial function ^41^: it has therefore been suggested that telomere attrition may impact mitochondrial activity, thus leading to metabolic dysregulation ^42^. In animal models, such mitochondrial dysfunction may manifest as increased adiposity and insulin resistance ^43^. In this latter case, the causal direction could be reversed, with telomere attrition as a risk factor for disease. However, a Mendelian randomisation analysis (in which genetic variants are used as non-confounded instrumental variables of disease risk factors ^44^), of telomere length in relation to BMI found no association in this direction ^3^.

Aviv and colleagues challenge the telomere clock hypothesis by suggesting “that individuals who are born with relatively short telomeres tend to enter adulthood with short leucocyte telomere length” ^40^. Moreover, this group have observed that the variation in neonatal telomere length is larger than the average amount of attrition that would be expected over a lifetime. This challenges the clock hypothesis, since, if true, individuals should begin life with a ‘clock time’ of zero ^40^. Therefore, an alternate hypothesis is that telomere length is largely pre-determined at birth ^45^, and that variable rates of attrition in adulthood would not necessarily be enough to alter an individual’s telomere length percentile ranking ^40^. Whilst this does not negate the possibility that oxidative stress later in life may still contribute to attrition, this group state that early determinants of telomere length may be more important ^45^ Under this assumption, combining estimates of neonatal adiposity in relation to telomere length ascertained at different ages should not alter results appreciably, as each individual would be placed on a set trajectory, altered little by postnatal exposures. In this case, it could be postulated that neonatal adiposity would have a greater association with telomere length than postnatal adiposity measures (including childhood adiposity). However, our results do not provide evidence for this hypothesis, since we found no strong evidence for an association between either neonatal adiposity with telomere length.

### Strengths and limitations

Although the relationship between adiposity and telomere length has been studied previously ^7, 8^, to our knowledge, this is the first study to systematically review and meta-analyse the evidence concerning neonatal adiposity measures and telomere length. However, there are a number of limitations to this work. Firstly, although we found 19 meta-analysable estimates, the differing study designs meant that estimates were only combinable in small groups, and 13 estimates were not combinable at all. Thus, power to detect associations within each individual category (most of which meta-analysed only 2-3 estimates in each) was limited. Where possible, we contacted authors to obtain the necessary information to standardise estimates, permitting them to be included in the meta-analysis. However, many of the source publications were written over 15 years ago, and the original data were not available. The meta-analysis may be subject to non-inclusion bias if the studies included in the meta-analyses are different to those not included. That said, we performed a narrative synthesis of those estimates which we were unable to include in the meta-analyses and conclusions were largely the same. The small number of studies retrieved, combined with their poor combinability, meant that meaningful inference from risk of bias assessments would not have been possible. Despite finding no strong evidence of non-inclusion bias, we acknowledge that publication bias remains a possibility, and this is therefore a limitation of our work. We were not able to make meaningful inferences about the likely presence of small-study effects using funnel plots, since there were so few combinable studies in each group ^46^. Not only did studies vary in the measures of adiposity studied (i.e. low birth weight versus small-for-gestational age as measures of neonatal adiposity), and whether they were studied as continuous or binary exposures, but studies also varied by method used to assay telomere length, as well as the transformations performed on exposure and outcome variables, and the age of the children studied. Most studies performed only minimal adjustment for potential confounding variables (or only adjusted exposures), thus we cannot rule out unmeasured or residual confounding. The lack of adjustment for prenatal factors in most studies also makes it difficult to establish whether the associations observed are due to a foetal predisposition to larger or smaller body size, or in utero effects. For example, birth weight may act as a surrogate marker for many maternal sources of in utero adversity ^47^, and it may be these mechanisms that are important in determining telomere length. A meta-analysis focussing specifically on these exposures would therefore be of value in the field. Although we did not find evidence of an effect in this study, a Mendelian randomization framework may prove useful for establishing whether there is a likely causal relationship between adiposity and telomere length. Although Haycock *et al.* (2017) found no evidence of association between telomere length (exposure) and BMI (outcome) ^3^, the reverse direction (adiposity→telomere length, as assessed in this review) has not been studied. Utilising the two-sample MR framework in order to assess adiposity as a causal determinant of telomere length would represent a highly powered method of assessing causality using summary-level genetic data.

We harmonised effect estimates into standardised units that would allow comparison of estimates obtained from both qPCR and TRF telomere lengths. However, whilst this allowed comparisons of telomere metrics measured on different scales, it does not address measurement error. Generally, Southern blot estimates (by TRF) may be longer than telomere length measured by qPCR due to inclusion of subtelomeric regions in the measure ^48^. Furthermore, there is evidence that different assays have different sensitivity to measuring extremes of telomere lengths, and as such the relationships between the two measures may be non-linear ^48, 49^. Quantitative PCR measurements (which relate the relative fluorescence of a telomere amplicon to a single-gene reference ^50^) have their own limitations, being more prone to inter and intra-assay variation than the gold standard measurement method of TRF analysis ^48, 51^. Whilst the majority of papers using qPCR reported coefficients of variation, suggesting an attempt to minimise batch effects had been made, the single-gene reference for qPCR assays varied between studies, which may have affected assay performance.

## Conclusions

We found no strong evidence of a relationship between either neonatal or childhood measures of adiposity and concurrently or prospectively measured telomere length, but there were few combinable studies, and amongst published studies there was substantial heterogeneity in observed effects. Further work is needed to clarify whether neonatal and childhood adiposity is associated with telomere length.

Abbreviations: BMI=Body Mass Index; WC=Waist Circumference; WHR=Waist-to-hip Ratio; qPCR=quantitative Polymerase Chain Reaction; TRF=Terminal Restriction Fragment; SMD=Standardised Mean Difference; PRISMA=Preferred Reporting Items for Systematic Reviews and Meta-Analyses

## Data availability

All data underlying the results are available as part of the article and supplementary material, and no additional source data are required.

## Supplementary material

**Supplementary File 1:** Search strategy.

**Supplementary File 2:** Collation of formulae used in data harmonisation and extraction.

**Supplementary File 3:** R script file used for performing harmonisation prior to analysis.

**Supplementary File 4:** Stata .do file used for performing meta-analysis.

**Supplementary Figure 1:** Flowchart detailing how abstracts were screened and coded.

**Supplementary Figure 2:** Flowchart detailing how full texts were screened and coded.

**Supplementary Figure 3:** PRISMA flowchart. Flowchart detailing the number of papers retrieved at the beginning of the screening process, and reasons for exclusion at each stage.

**Supplementary Figure 4**: Completed ‘MOOSE’ (Meta-analyses Of Observational Studies in Epidemiology) checklist.

**Supplementary Tables 1–3:** Summary of papers retrieved, Summary of reasons for exclusion at title/abstract stage and Summary of reasons for exclusion at full text stage.

**Supplementary Table 4:** Summary of papers assessed.
