# Supplementary material for: Early life adiposity and telomere length across the life course: a systematic review and meta-analysis
Source: Wellcome Open Res. 2018 Aug 7;2:118. Originally published 2017 Dec 18. [Version 2] doi: 10.12688/wellcomeopenres.13083.2 (PMC6259597; doi:10.12688/wellcomeopenres.13083.2)
Supplement: Supplementary file 1 [file wellcomeopenres-2-16039-s0000.tgz › 237462c6-b235-4f0f-a063-f9a76f9fc51e.pdf]

Supplementary File 1

Search strategy (22042017):

Research question to be answered: What is the relationship between measures of childhood adiposity and telomere length, either cross-sectionally or longitudinally?

Medline and EMBASE (using the Ovid platform), as well as PubMed were searched. The search strategies are detailed as below:

Medline (in process 1950-present) (Telo-Adiposity SR MEDLINE, **Ovid MEDLINE(R)** 1946 to present)

1. Adiposity/
2. body mass index/
3. exp Body Weight/
4. exp Waist Circumference/
5. waist-height ratio/
6. Waist-Hip Ratio/
7. Skinfold Thickness/
8. adipos*.ti,ab.
9. bmi.ti,ab.
10. body mass index.ti,ab.
11. overweight.ti,ab.
12. waist circumference.ti,ab.
13. waist hip ratio.ti,ab.
14. waist height ratio.ti,ab.
15. skinfold thickness.ti,ab.
16. obes*.ti,ab.
17. birthweight.ti,ab.
18. birth weight.ti,ab.
19. weight.ti,ab
20. body fat.ti,ab.
21. fat mass.ti,ab.
22. fatness.ti,ab
23. waist to hip ratio.ti,ab.
24. waist to height ratio.ti,ab.
25. abdominal circumference.ti,ab
26. ponderal ind*.ti,ab
27. Telomere/
28. Telomere Shortening/
29. Telomere Homeostasis/
30. telomere.ti,ab.
31. child.ti,ab.
32. children.ti,ab.
33. paediatric.ti,ab.
34. pediatric.ti,ab.
35. adolescen*.ti,ab.

36. teen*.ti,ab
37. boy*.ti,ab
38. girl*.tiab
39. newborn*.ti,ab
40. (new adj born).ti,ab.
41. neonat*.ti,ab.
42. baby.ti,ab.
43. babies.ti,ab.
44. infan*.ti,ab.
45. infant/
46. child/
47. adolescent/
48. 1 or 2 or 3 or 4 or 5 or 6 or 7 or 8 or 9 or 10 or 11 or 12 or 13 or 14 or 15 or 16 or 17 or 18 or 19 or 20 or 21 or 22 or 23 or 24 or 25 or 26
49. 27 or 28 or 29 or 30
50. 31 or 32 or 33 or 34 or 35 or 36 or 37 or 38 or 39 or 40 or 41 or 42 or 43 or 44 or 45 or 46 or 47
51. 48 and 49 and 50

EMBASE 1980 to 2015 Week 26 (Telo-Adiposity SR EMBASE, **Embase** 1974 to 2017 Week 17)

1. obesity/
2. body mass/
3. exp body weight/
4. waist circumference/
5. waist to height ratio/
6. waist hip ratio/
7. skinfold thickness/
8. adipos*.ti,ab.
9. bmi.ti,ab.
10. body mass index.ti,ab.
11. overweight.ti,ab.
12. waist circumference.ti,ab.
13. waist hip ratio.ti,ab.
14. waist height ratio.ti,ab.
15. skinfold thickness.ti,ab.
16. obes*.ti,ab.
17. birthweight.ti,ab.
18. birth weight.ti,ab.
19. weight.ti,ab
20. body fat.ti,ab.
21. fat mass.ti,ab.
22. fatness.ti,ab
23. waist to hip ratio.ti,ab.
24. waist to height ratio.ti,ab.

25.	abdominal circumference.ti,ab
26.	ponderal ind*.ti,ab
27.	telomere/
28.	telomere shortening/
29.	telomere homeostasis/
30.	telomere.ti,ab.
31.	child.ti,ab.
32.	children.ti,ab.
33.	paediatric.ti,ab.
34.	pediatric.ti,ab.
35.	adolescen*.ti,ab.
36.	teen*.ti,ab
37.	boy*.ti,ab
38.	girl*.tiab
39.	newborn*.ti,ab
40.	(new adj born).ti,ab.
41.	neonat*.ti,ab.
42.	baby.ti,ab.
43.	babies.ti,ab.
44.	infan*.ti,ab.
45.	infant/
46.	child/
47.	adolescent/
48.	1 or 2 or 3 or 4 or 5 or 6 or 7 or 8 or 9 or 10 or 11 or 12 or 13 or 14 or 15 or 16 or 17 or 18 or 19 or 20 or 21 or 22 or 23 or 24 or 25 or 26
49.	27 or 28 or 29 or 30
50.	31 or 32 or 33 or 34 or 35 or 36 or 37 or 38 or 39 or 40 or 41 or 42 or 43 or 44 or 45 or 46 or 47
51.	48 and 49 and 50

PubMed

PubMed (Jan2016_asJuly_plus_infan_textterm)

1.	Adiposity[mesh:noexp]
2.	body mass index[mesh:noexp]
3.	Body Weight[Mesh]
4.	Waist Circumference[Mesh]
5.	waist-height ratio[mesh:noexp]
6.	Waist-Hip Ratio[mesh:noexp]
7.	Skinfold Thickness[mesh:noexp]
8.	adipos*[tiab]
9.	bmi[tiab]
10.	body mass index[tiab]

11. overweight[tiab]
12. waist circumference[tiab]
13. waist hip ratio[tiab]
14. waist height ratio[tiab]
15. skinfold thickness[tiab]
16. obes*[tiab]
17. birthweight[tiab]
18. birth weight[tiab]
19. weight[tiab]
20. body fat[tiab]
21. fat mass[tiab]
22. fatness[tiab]
23. waist to hip ratio[tiab]
24. waist to height ratio[tiab])
25. abdominal circumference[tiab]
26. ponderal ind*[tiab]
27. Telomere[mesh:noexp]
28. Telomere Shortening[mesh:noexp]
29. Telomere Homeostasis[mesh:noexp]
30. telomere[tiab]
31. child[tiab]
32. children[tiab]
33. paediatric[tiab]
34. pediatric[tiab]
35. adolescen*[tiab]
36. teen*[tiab]
37. boy*[tiab]
38. girl*[tiab]
39. newborn*[tiab]
40. “new born”[tiab]
41. neonat*[tiab]
42. baby[tiab]
43. babies[tiab]
44. infant*[tiab]
45. infant[Mesh]
46. child[Mesh]
47. adolescent[Mesh]
48. 1 or 2 or 3 or 4 or 5 or 6 or 7 or 8 or 9 or 10 or 11 or 12 or 13 or 14 or 15 or 16 or 17 or 18 or 19 or 20 or 21 or 22 or 23 or 24 or 25 or 26
49. 27 or 28 or 29 or 30
50. 31 or 32 or 33 or 34 or 35 or 36 or 37 or 38 or 39 or 40 or 41 or 42 or 43 or 44 or 45 or 46 or 47
51. 48 and 49 and 50
