# Supplementary material for: Early life adiposity and telomere length across the life course: a systematic review and meta-analysis
Source: Wellcome Open Res. 2018 Aug 7;2:118. Originally published 2017 Dec 18. [Version 2] doi: 10.12688/wellcomeopenres.13083.2 (PMC6259597; doi:10.12688/wellcomeopenres.13083.2)
Supplement: Supplementary file 2 [file wellcomeopenres-2-16039-s0001.tgz › 0bf024d1-27f1-43e5-920d-c4f8a46d91e4.pdf]

Supplementary File 2: Collation of formulae used in data harmonisation and extraction

Standard error (SEM) from standard deviation (SD):

$$SEM = \frac{SD}{\sqrt{N}}$$

Pooled standard deviation:

$$SD_{pooled} = \sqrt{\frac{(n_1 - 1)SD_1^2 + (n_2 - 1)SD_2^2}{n_1 + n_2 - 2}}$$

Standard error of the mean difference (SEMD):

$$SEMD = \sqrt{\frac{SD_1^2}{n_1} + \frac{SD_2^2}{n_2}}$$

See: http://vassarstats.net/dist2.html

Convert mean difference to standardised mean difference (SMD):

$$SMD = \frac{Difference\ in\ mean\ outcome\ between\ groups}{Standard\ deviation\ of\ outcome\ among\ participants}$$

See: http://handbook.cochrane.org/chapter_9/9_2_3_2_the_standardized_mean_difference.htm

Standard error of the standardised mean difference (d):

$$SE_d = \sqrt{\frac{n_1 + n_2}{n_1 n_2} + \frac{d^2}{2(n_1 + n_2)}}$$

See: https://www.meta-analysis.com/downloads/Meta-analysis%20Effect%20sizes%20based%20on%20means.pdf

Unstandardised beta (β_{std}) to standardised beta (β_{unstd}):

$$\beta_{std} = \beta_{unstd}\left(\frac{SD_{exposure}}{SD_{outcome}}\right)$$

See: http://www.albany.edu/faculty/kretheme/PAD705/SupportMat/StandardizedCeof.pdf

t-statistic from β and SEM:

$$t = \frac{\beta_{unstd}}{SEM_{unstd}} = \frac{\beta_{std}}{SEM_{std}}$$

…and therefore, can find SEM_{std} if know β_{unstd}, SEM_{unstd} and β_{std}:

$$SEM_{std} = \frac{\beta_{std}}{t}$$
