# Supplementary figures and images for: Early life adiposity and telomere length across the life course: a systematic review and meta-analysis
Source: Wellcome Open Res. 2018 Aug 7;2:118. Originally published 2017 Dec 18. [Version 2] doi: 10.12688/wellcomeopenres.13083.2 (PMC6259597; doi:10.12688/wellcomeopenres.13083.2)

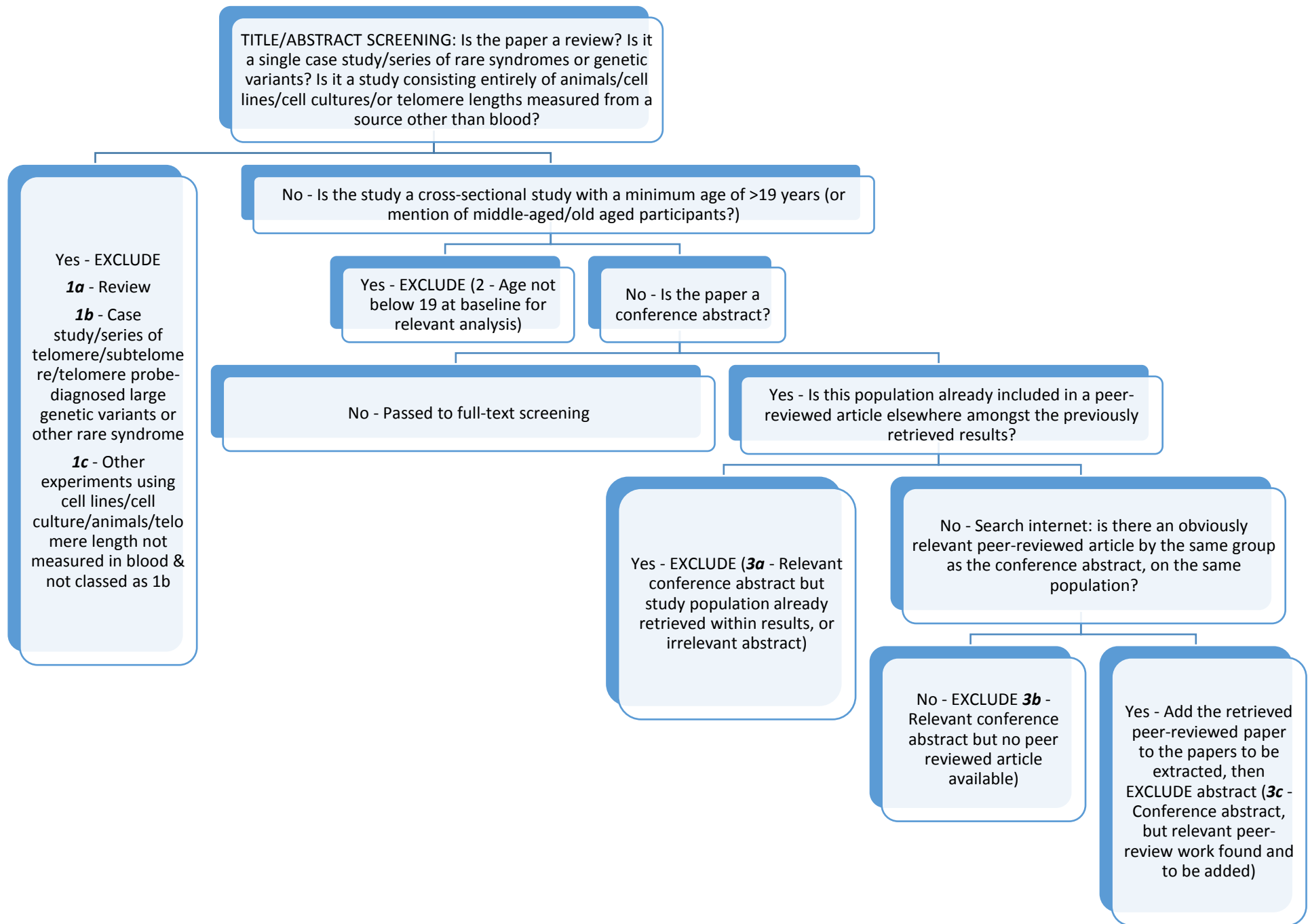

Supplement: Supplementary file 5 [file wellcomeopenres-2-16039-s0004.tgz › 41fcaa2c-2068-4934-9177-d175c326262e.pdf]

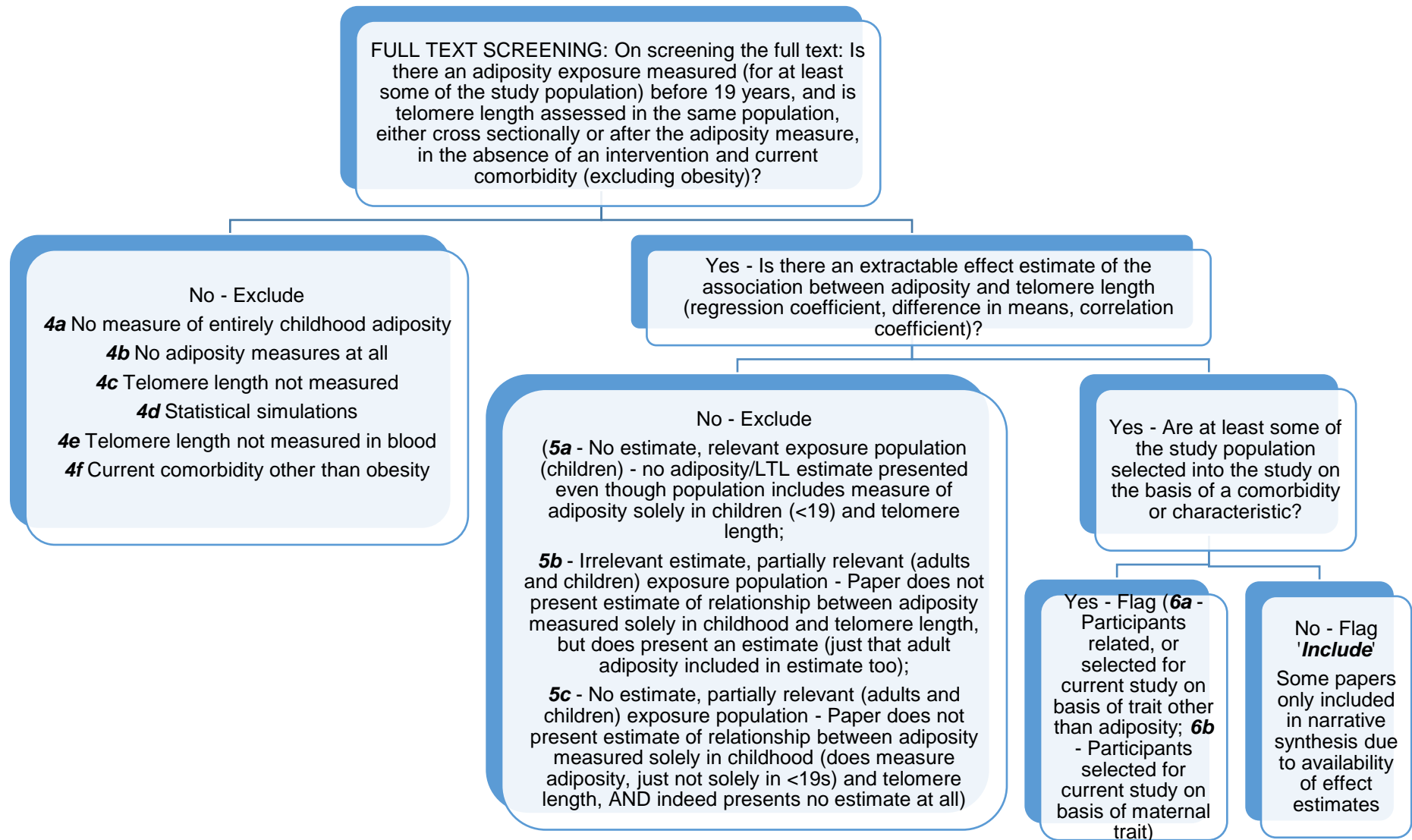

Supplement: Supplementary file 6 [file wellcomeopenres-2-16039-s0005.tgz › ca5336bc-5eda-4bbd-849f-17ca4a581840.pdf]
