# Supplementary material for: Early life adiposity and telomere length across the life course: a systematic review and meta-analysis
Source: Wellcome Open Res. 2018 Aug 7;2:118. Originally published 2017 Dec 18. [Version 2] doi: 10.12688/wellcomeopenres.13083.2 (PMC6259597; doi:10.12688/wellcomeopenres.13083.2)

Supplementary Figure 3: PRISMA flowchart

Flowchart detailing the number of papers retrieved at the beginning of the screening process, and reasons for exclusion at each stage.

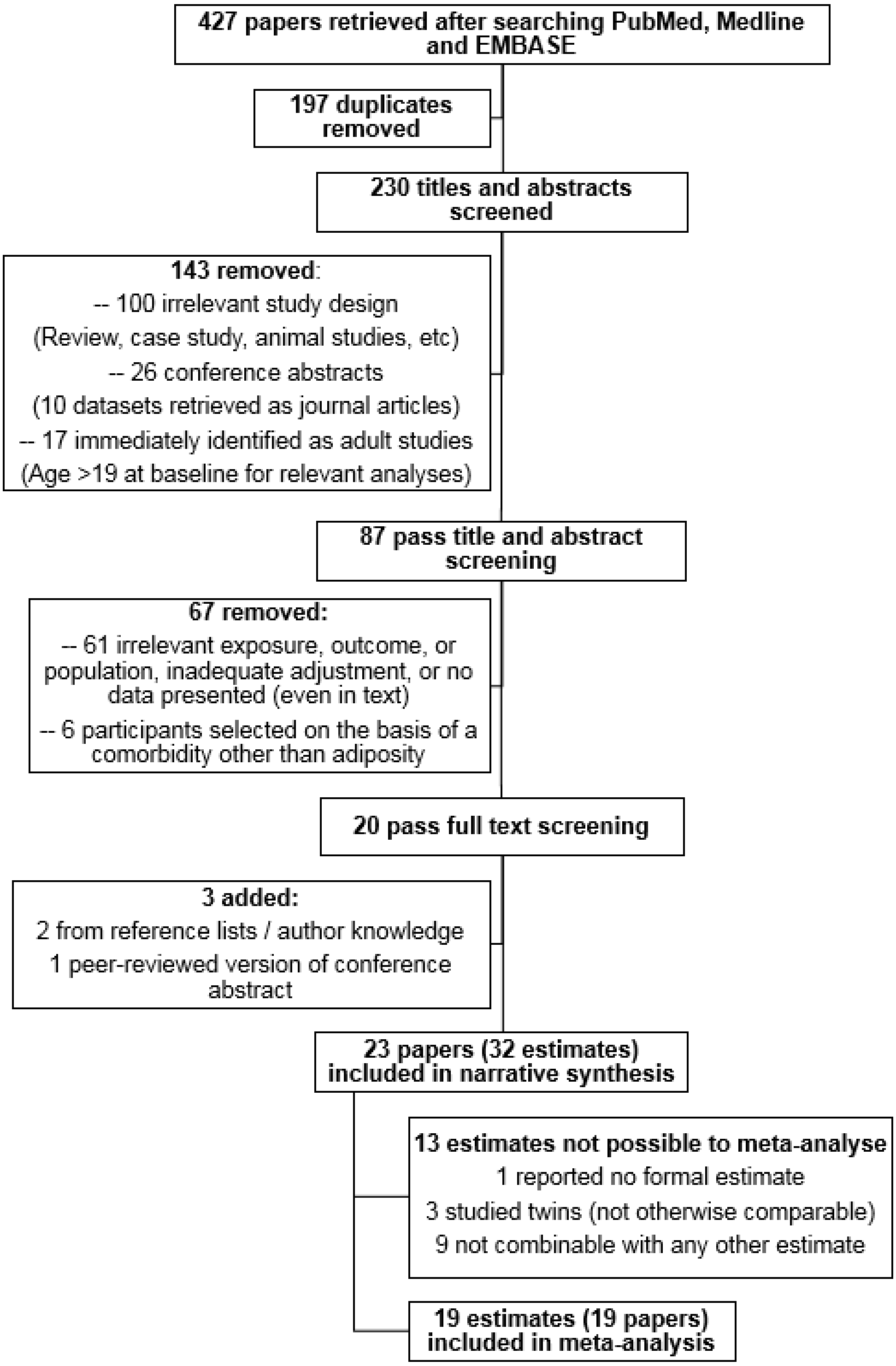

Supplement: Supplementary file 7 [file wellcomeopenres-2-16039-s0006.tgz › 5473984d-58b0-4ae5-aff9-e2ffcea4e4b7.pdf]
