# Supplementary material for: Early life adiposity and telomere length across the life course: a systematic review and meta-analysis
Source: Wellcome Open Res. 2018 Aug 7;2:118. Originally published 2017 Dec 18. [Version 2] doi: 10.12688/wellcomeopenres.13083.2 (PMC6259597; doi:10.12688/wellcomeopenres.13083.2)
Supplement: Supplementary file 9 [file wellcomeopenres-2-16039-s0008.tgz › c28ea39b-2beb-4021-bbf1-193d43ec775b.docx]

Supplementary Table 1: Summary of papers retrieved

Database	#Papers
Pubmed	116
Medline	109
Embase	202

Search date: 220417

Search ran as above.

Imported to EndNote

230 papers **for assessment** (197 duplicates identified)

Supplementary Table 2: Summary of reasons for exclusion at title and abstract stage

**Category**	**Code**	**#**	**Code description**
Irrelevant	1a	26	Review
	1b	48	Case study/series of telomere/subtelomere/telomere probe-diagnosed large genetic variants or other rare syndrome
	1c	26	Other experiments using cell lines/cell culture/animals/telomere length not measured in blood & not classed as 1b
Irrelevant population (from abstract)	2	17	Age not below 19 at baseline for relevant analysis
Conference abstract	3a	10	Relevant conference abstract but study population already retrieved within results, or irrelevant abstract
	3b	15	Relevant conference abstract but no peer reviewed article available
	3c	1	Conference abstract, but relevant peer-review work found and to be added
Relevant	‘Passed’	87	Title/abstract passed initial screening
	**TOTAL**	**230**	

Supplementary Table 3: Summary of reasons for exclusion at full text stage

Category	Exclusion Code	#	Description
Exposure and or outcome missing on closer inspection	4a	10	No measure of entirely childhood adiposity
	4b	3	No adiposity measures at all
	4c	4	Telomere length not measured
	4d	1	Statistical simulations
	4e	1	Telomere length not measured in blood
	4f	2	Current comorbidity other than obesity
Issue with estimates provided	5a	13	No estimate, relevant exposure population (children) - no adiposity/LTL estimate presented even though population includes measure of adiposity solely in children (<19) and telomere length
	5b	14	Irrelevant estimate, partially relevant (adults and children) exposure population - Paper does not present estimate of relationship between adiposity measured solely in childhood and telomere length, but does present an estimate (just that adult adiposity included in estimate too)
	5c	13	No estimate, partially relevant (adults and children) exposure population - Paper does not present estimate of relationship between adiposity measured solely in childhood (does measure adiposity, just not solely in <19s) and telomere length, AND indeed presents no estimate at all)
Selected on criteria (not adiposity)	6a	4	Participants related or selected for current study on basis of trait other than adiposity
	6b	2	Participants selected for current study on basis of maternal trait
Total excluded		**67/87**	
Total included at this stage (April 2017)		**20/87**	Title/abstract passed full text screening from original search
Additions (until April 2017)		1	Full text of retrieved conference abstract (Bethancourt)
		2	Authors' knowledge / after searching reference lists (Masi, Davy)
**TOTAL INCLUDED**		**23**	**NB 4 papers not included in meta-analysis (Okuda, Bethancourt, Buxton [2014], Strohmaier)**
