# Supplementary material for: Early life adiposity and telomere length across the life course: a systematic review and meta-analysis
Source: Wellcome Open Res. 2018 Aug 7;2:118. Originally published 2017 Dec 18. [Version 2] doi: 10.12688/wellcomeopenres.13083.2 (PMC6259597; doi:10.12688/wellcomeopenres.13083.2)
Supplement: Peer review history

# Peer review: Referee response for version 2

**DOI:** 10.21956/wellcomeopenres.16039.r33645

**Published:** 2018-11-23

**Authors:** Mariana L. Tellechea

**Affiliations:** 1 Institute of Medical Research (IDIM), National Scientific and Technical Research Council (CONICET), Buenos Aires University (UBA), Buenos Aires, Argentina

Authors have addressed most of the issues indicated in the review report and I believe that the revised version can now meet the journal publication requirements.

 In reference to item 4 (minor concerns) I have suggested that owning to the importance of confounders age and gender in telomere biology, description of adjustment for age and sex should have had a main role in methods section.

I have read this submission. I believe that I have an appropriate level of expertise to confirm that it is of an acceptable scientific standard.

# Peer review: Referee response for version 2

**DOI:** 10.21956/wellcomeopenres.16039.r33646

**Published:** 2018-08-22

**Authors:** Eduardo Mundstock, Edgar Sarria, Rita Mattiello

**Affiliations:** 1 Programa de Pós-Graduação em Pediatria e Saúde da Criança, Pontifícia Universidade Católica do Rio Grande do Sul (PUCRS), Porto Alegre, Brazil; 2 Programa Esporte e Saúde em Canela; Secretaria de Educação, Esporte e Lazer de Canela/RS (SMEEL), Canela, Brazil; 3 Programa de Pós-graduação em Medicina e Ciências da Saúde, Pontifícia Universidade Católica do Rio Grande do Sul (PUCRS), Porto Alegre, Brazil; 4 Programa de Pós-graduação em Epidemiologia, Universidade Federal do Rio Grande do Sul (UFRGS), Porto Alegre, Brazil; 5 Departamento de Biologia e Farmácia, Curso de Medicina, Universidade de Santa Cruz do Sul (UNISC), Santa Cruz do Sul, Brazil

Authors' responses were satisfactory

We have read this submission. We believe that we have an appropriate level of expertise to confirm that it is of an acceptable scientific standard.

# Peer review: Referee response for version 1

**DOI:** 10.21956/wellcomeopenres.14189.r33068

**Published:** 2018-05-11

**Authors:** Eduardo Mundstock, Edgar Sarria, Rita Mattiello

**Affiliations:** 1 Programa de Pós-Graduação em Pediatria e Saúde da Criança, Pontifícia Universidade Católica do Rio Grande do Sul (PUCRS), Porto Alegre, Brazil; 2 Programa Esporte e Saúde em Canela; Secretaria de Educação, Esporte e Lazer de Canela/RS (SMEEL), Canela, Brazil; 3 Programa de Pós-graduação em Medicina e Ciências da Saúde, Pontifícia Universidade Católica do Rio Grande do Sul (PUCRS), Porto Alegre, Brazil; 4 Programa de Pós-graduação em Epidemiologia, Universidade Federal do Rio Grande do Sul (UFRGS), Porto Alegre, Brazil; 5 Departamento de Biologia e Farmácia, Curso de Medicina, Universidade de Santa Cruz do Sul (UNISC), Santa Cruz do Sul, Brazil

Authors aimed to systematically review and meta-analyze the results of studies assessing associations between early in life (neonatal / childhood) adiposity and telomere length

 The article is well written and contains important and original information. Below I leave some questions, besides those already carried out by the other reviewers, that I believe should be answered by the authors.

 The search is over one year old. It may be interesting (not mandatory) to redo it. In addition the search was carried out in only 3 databases, although they are important bases, bases like the web the science and Scopus could be consulted.

 The selection process by summary and title was performed by a single author, although this does not prevent the publication of the article, I suggest that in future reviews the authors follow the Cochrane recommendation “Assessment of eligibility of studies, and extraction of data from study reports, should be done by at least two people, independently”. (Chapter 7:  Selecting studies and collecting data) ^1^

 The text below would be better placed in the results, in the methodology you should describe how the data were treated.

 "There was considerable heterogeneity in the presentation of findings across studies (Table 1 and Table 2). Some studies22-24 only presented differences in the form of bar charts "

 There was record of the protocol in some base (preferably in PROSPERO). If not, I strongly suggest that the protocol be recorded in future reviews.

 A total of 427 papers that were published until April 22, 2017 were obtained after searching Medline, EMBASE and PubMed (Supplementary Figure 3). **A total of 230 titles remained for assessment (What are the reasons for excluding these 197 articles duplicates? Not eligible and why? Etc).**

 In addition to the analysis of the risk of publication bias, already mentioned by the other reviewers, the authors did not perform the analysis of risk of bias within the studies.

 The authors declare in the discussion **"The small number of studies retrieved, combined with their poor combinability, meant that meaningful inference from risk of bias assessments would not have been possible"** but the analysis of the risk of bias within the studies is conducted in each study individually so, the authors can use some tool for this analysis (or at least for quality analysis of each study).

We have read this submission. We believe that we have an appropriate level of expertise to confirm that it is of an acceptable scientific standard, however we have significant reservations, as outlined above.

## References

[1] HigginsJPT: Cochrane Handbook for Systematic Reviews of Interventions Version 5.1.0 [updated March 2011]. *The Cochrane Collaboration*.2011; Reference source

# Response

**Published:** 2018-07-31

**Authors:** Anna Guyatt

**General response:**

 Again, we would like to thank the reviewer for their encouragement and advice on our manuscript. We have responded to each of the queries in turn, hereafter.

**Authors aimed to systematically review and meta-analyze the results of studies assessing associations between early in life (neonatal / childhood) adiposity and telomere length**

****

**The article is well written and contains important and original information. Below I leave some questions, besides those already carried out by the other reviewers, that I believe should be answered by the authors.**

****

**The search is over one year old. It may be interesting (not mandatory) to redo it. In addition the search was carried out in only 3 databases, although they are important bases, bases like the web the science and Scopus could be consulted.**

****

**The selection process by summary and title was performed by a single author, although this does not prevent the publication of the article, I suggest that in future reviews the authors follow the Cochrane recommendation “Assessment of eligibility of studies, and extraction of data from study reports, should be done by at least two people, independently”. (Chapter 7:  Selecting studies and collecting data) ^1^**

*Response: We thank the reviewer for their pragmatism. As is inevitable with any systematic review, there comes a point where it is not feasible to keep re-running the searches (and thus re-running multiple sets of analyses) and to publish the paper. We have therefore been clear about the date when the search was last conducted. There is considerable overlap between the searchable databases, and given the substantial number of duplicates we retrieved in our literature search, we do not believe that interrogating additional databases would be likely to add to the number of papers retrieved, especially as we hand-searched reference lists to capture additional studies for inclusion.*

*Given that this systematic review is not a Cochrane Review specifically, we deemed it appropriate that one author screen for any potentially eligible studies that might be included. The author was very inclusive at this initial stage, bringing forward any paper that appeared to be potentially eligible for inclusion. Moreover, as mentioned above, additional papers were picked up in the searches of all reference lists for included papers (which was conducted by two independent authors). We feel it is very likely that any papers missed by the author conducting the initial ‘eligibility’ screening would have been later picked up in the reference lists of included studies.*

**The text below would be better placed in the results, in the methodology you should describe how the data were treated.**

**"There was considerable heterogeneity in the presentation of findings across studies (Table 1 and Table 2). Some studies22-24 only presented differences in the form of bar charts "**

*Response: Whilst we appreciate that this sentence may initially read as Results and not Methods, we were explaining the reasons for standardising the estimates rather than commenting on empirical heterogeneity (which is in the Results section). For clarity, we have rewritten it as follows:*

*Results: “To facilitate the pooling of results according to different transformations of both exposures and outcomes (e.g. normalisation, z-scoring, log-transformation), all estimates were standardised for the meta-analyses. Plot digitiser software [http://arohatgi.info/WebPlotDigitizer] was used to extract data from studies presenting differences in means in the form of bar charts.”*

**There was record of the protocol in some base (preferably in PROSPERO). If not, I strongly suggest that the protocol be recorded in future reviews.**

*Response:* *We thank the reviewer for their suggestion.*

**A total of 427 papers that were published until April 22, 2017 were obtained after searching Medline, EMBASE and PubMed (Supplementary Figure 3). A total of 230 titles remained for assessment (What are the reasons for excluding these 197 articles duplicates? Not eligible and why? Etc).**

*Response: These 197 articles were excluded because on they were exact duplicates of some of the 230 articles that passed to screening (i.e. they were picked up by multiple databases).*

**In addition to the analysis of the risk of publication bias, already mentioned by the other reviewers, the authors did not perform the analysis of risk of bias within the studies.**

**The authors declare in the discussion "The small number of studies retrieved, combined with their poor combinability, meant that meaningful inference from risk of bias assessments would not have been possible" but the analysis of the risk of bias within the studies is conducted in each study individually so, the authors can use some tool for this analysis (or at least for quality analysis of each study).**

*Response: We thank the reviewer for their suggestion. We note that risk of bias within studies is extremely subjective. Tools for assessing quality in clinical trials are well-described but much less attention has been given to similar tools for observational epidemiological studies. Thus, formally examining bias within observational studies is not feasible, and there is no gold standard tool for its assessment. We refer the reviewer to the following systematic review of tools for assessing bias in observational studies (https://www.ncbi.nlm.nih.gov/pubmed/17470488), wherein the authors quote:*

 “ *This review has highlighted the lack of a single obvious candidate tool for assessing quality of observational epidemiological studies. One might regard this review as the first stage towards development of a generic tool. In such an endeavour, one would need to reach a consensus on the critical domains that should be included. The development of the STROBE statement has involved extensive discussion among numerous experienced epidemiologists and statisticians. Despite targeting the reporting of studies, many items were no doubt selected due to presumed (or evidence of) association with susceptibility to bias. Thus the statement should provide a suitable starting point for development of a quality assessment tool, and we have been guided by it in our presentation of results*.”

# Peer review: Referee response for version 1

**DOI:** 10.21956/wellcomeopenres.14189.r30206

**Published:** 2018-02-08

**Authors:** Mariana L. Tellechea, Adriana Burgueño

**Affiliations:** 1 Institute of Medical Research (IDIM), National Scientific and Technical Research Council (CONICET), Buenos Aires University (UBA), Buenos Aires, Argentina; 2 Institute of Medical Investigation (BIOMED), National Scientific and Technical Research Council (CONICET), Buenos Aires, Argentina

Authors aimed to systematically review and meta-analyze the results of studies assessing associations between early in life (neonatal/childhood) adiposity and telomere length. Recently (on years 2014, 2015) other authors had reported on evidence from studies of adiposity in childhood, but this is the first meta-analysis of adiposity and telomere length to synthesize evidence from neonatal measures.

 This referee thinks that the issue is well prepared and updated. All tables and figures are clear and the references seem appropriate. Supporting material is also suitable enough; authors also presented the “Stata.do” file used for performing meta-analysis.

 I believe that this issue will be a contribution to the readers of this journal and I would recommend the paper for indexing after some changes.

 The main limitation I find is mentioned by the authors in the Discussion section. Studies on the association between the telomere length and adiposity in the neonatal period are not scarce (n= 11), however the estimates were only combinable in small groups (n= 2, n= 3, n= 4). Studies vary not only in the measures of adiposity studied but also whether they were studied as continuous or binary exposures. Each separate meta-analysis includes small number of studies, and the power of the test in such circumstances is low.

 Other major considerations should be taken into account. I would suggest a restructuring of the manuscript (mainly from the Methods section), and perhaps also changing the manuscript Title.

1. Some included studies were aimed to assess the difference in telomere length between small-for-gestational-age and appropriate-for-gestational age (or appropriate- and large-for-gestational age). Even a dataset was extracted from a manuscript on faetal growth retardation (Davy 2009, Table 2):
  It has been previously described that maternal complications (and even the low or high birth weight) would be related to complications in adult life. Moreover, the authors in the Discussion section mention that early determinants of telomere length may be important. I would suggest that a meta-analysis based on data on perinatal complications be considered separately, mainly because the underling mechanisms could be different. According, authors should dedicate a paragraph in the Discussion section to expand this topic.
  Regarding the meta-analysis “AGA vs. SGA (Difference in TL [SD])”, it should be also noted that even if effect sizes were expressed as the difference in telomere length between the two groups, those estimates are not measures of “adiposity”.
2. Authors included both cross-sectional studies (in which adiposity and telomere length were measured concurrently) and longitudinal studies (in which adiposity was measured in the neonatal period/childhood and telomere length was measured later in childhood or adulthood):
  Although the authors performed two separate meta-analyzes, it would be convenient to explain in Methods section that two analyzes with different specific objectives will be carried out, because I believe that the interpretation of the results of one or another study may not be the same. For longitudinal studies, the authors do not established an age of telomere length measurement. Therefore, it would be interesting for the reader to have a brief discussion about the aim of this specific study. I believe that the manuscript would be more valuable if data on “shortening” of telomere length is analyzed. Is it possible to add this analysis with the data provided in the manuscripts? Is it possible to request this information from the authors of the longitudinal studies?
3. To facilitate the pooling of results, all estimates were standardized:
  Perhaps in order to diminish a possible heterogeneity on datasets, the raw data could have been requested to original authors. Besides, this could have increased the number of included studies.
4. Potential “Publication bias” assessment is missing in the manuscript. 

 

 Minor concerns:

  

1. In the Discussion section, the authors mention that heterogeneity is observed in the meta-analysis of categorical variables, and that this could be due to the cut-off values to define groups. However, at this point the authors should also mention other potential causes of heterogeneity such as the telomere length detection method, ethnicity, etc. Moreover, most studies performed adjustment for potential confounding variables age and sex, but we cannot exclude unmeasured or residual confounding. It is also possible that perinatal factors were involved. For example, data on Entringer 2013 manuscript was corrected for “Obstetric complications, preg. Specific stress” (Table 2), and although in this dataset the estimate was corrected, we cannot assume that other studies were free of perinatal complications.
2. In the Methods section, it is not clearly explained that different meta-analysis will be carried out. I would establish a priori that different studies will be done, and according to this, different types of data will be collected. For example, different measurements of “adiposity” were considered for incorporation into the meta-analysis, including weight and birth weight, but weight or birth weight are not measures of adiposity by themselves. Some studies presented estimates of average telomere length by adiposity exposure groups, for example, in obese vs. non obese, and such studies are eligible indeed.
3. Perhaps the authors should add a brief paragraph on the meaning of the measurement of telomere length in peripheral blood or cord, instead of the adipose tissue itself. 
4. Obeying the fact that the telomere length varies by age and gender, the paragraph on adjustment for age and sex in Methods section, should be written aside.
5. Eligible studies included those with at least one measure of adiposity in the neonatal period or childhood (mean age <19 years). Childhood is the age span ranging from birth to adolescence. Perhaps the authors should reconsider the name of the group or the age range.
6. Please add in the Tables the n of the groups (binary exposures). 
7. In Table 2, I was not able to find the meaning of the letters (A to F) in the Analysis column.
8. In Table 2, in the column "Sex Adjustment", in row 4 (De Zegher 2016) and also in row 16 (Shalev 2014) there is a question mark of uncertain significance. 
9. In Figures 1 and 2 the statistics I2 and the p-values are shown. I assumed that the p-value corresponds to the analysis of heterogeneity, but it is actually not clear.
10. In Figures 1 and 2 it would be useful for the reader to find a brief explanation on how to read the Forest plot.

We have read this submission. We believe that we have an appropriate level of expertise to confirm that it is of an acceptable scientific standard, however we have significant reservations, as outlined above.

# Response

**Published:** 2018-07-31

**Authors:** Anna Guyatt

**General Response:** *We thank the reviewers for their detailed and constructive comments, which have improved our manuscript. We have responded to each point in turn below, explaining how we have addressed their concerns, and given details of where we have modified the manuscript accordingly.*

**Authors aimed to systematically review and meta-analyze the results of studies assessing associations between early in life (neonatal/childhood) adiposity and telomere length. Recently (on years 2014, 2015) other authors had reported on evidence from studies of adiposity in childhood, but this is the first meta-analysis of adiposity and telomere length to synthesize evidence from neonatal measures.**

****

**This referee thinks that the issue is well prepared and updated. All tables and figures are clear and the references seem appropriate. Supporting material is also suitable enough; authors also presented the “Stata.do” file used for performing meta-analysis.**

****

**I believe that this issue will be a contribution to the readers of this journal and I would recommend the paper for indexing after some changes.**

****

**The main limitation I find is mentioned by the authors in the Discussion section. Studies on the association between the telomere length and adiposity in the neonatal period are not scarce (n= 11), however the estimates were only combinable in small groups (n= 2, n= 3, n= 4). Studies vary not only in the measures of adiposity studied but also whether they were studied as continuous or binary exposures. Each separate meta-analysis includes small number of studies, and the power of the test in such circumstances is low.**

****

**Other major considerations should be taken into account. I would suggest a restructuring of the manuscript (mainly from the Methods section), and perhaps also changing the manuscript Title.**

****

**1.     Some included studies were aimed to assess the difference in telomere length between small-for-gestational-age and appropriate-for-gestational age (or appropriate- and large-for-gestational age). Even a dataset was extracted from a manuscript on faetal growth retardation (Davy 2009, Table 2):**

****

**It has been previously described that maternal complications (and even the low or high birth weight) would be related to complications in adult life. Moreover, the authors in the Discussion section mention that early determinants of telomere length may be important. I would suggest that a meta-analysis based on data on perinatal complications be considered separately, mainly because the underling mechanisms could be different. According, authors should dedicate a paragraph in the Discussion section to expand this topic.**

*Response: We thank the reviewers for raising this important point, which we have expanded upon in the Discussion.* *We think that the question of the relationship between perinatal complications and telomere length is an interesting question in its own right, and one that would merit discussion in a separate paper. Our paper focuses on measures of adiposity, or measures that act as proxies for adiposity (SGA and AGA) in early life.* *We did choose to include one study on foetal growth restriction (FGR)—Davy et al. (2009). We acknowledge that FGR in particular is associated with prenatal adversity. However, the study in question defined FGR simply as ‘as any newborn having a birth weight of ≤5th percentile for Filipino newborns at a given gestational age’. Therefore, although the nomenclature of the study referred to these newborns as being ‘FGR’, definition of FGR in this study is similar to SGA, albeit with a more extreme cut-off value than is often used. Anthropometric measures are not reported to have been undertaken serially in utero when defining FGR in this study. We therefore believe that inclusion of this study with the other studies of SGA and AGA is justified. We acknowledge the use of different cut-offs for defining binary exposures of body size/adiposity as a general limitation of this study, which is also pertinent to exposures measured later in childhood (i.e. BMI):*

*Discussion: “* *The lack of adjustment for prenatal factors in most studies also makes it difficult to establish whether the associations observed are due to a foetal predisposition to larger or smaller body size, or in utero effects. For example, birth weight may act as a surrogate marker for many maternal sources of in utero adversity (Tyrrell et al., 2016, https://www.ncbi.nlm.nih.gov/pubmed/26978208), and it may be these mechanisms that are important in determining telomere length. A meta-analysis focussing specifically on these exposures would therefore be of value in the field.”*

**Regarding the meta-analysis “AGA vs. SGA (Difference in TL [SD])”, it should be also noted that even if effect sizes were expressed as the difference in telomere length between the two groups, those estimates are not measures of “adiposity”.**

*Response: The main focus of our narrative review and SR is adiposity; after conducting the review, it was the case that the greatest number of neonatal papers focussed on birth weight adjusted for gestational age. We acknowledge that some indices capture adiposity better than others, but would argue that LGA and SGA are proxies for higher and lower adiposity levels, given that SGA is defined as “weight” below the 10 ^th^centile for gestational age. We note that a recent paper has reported a high correlation between weight and adiposity in neonates (Chen et al., 2018 https://www.ncbi.nlm.nih.gov/pubmed/28990589*).

**2.     Authors included both cross-sectional studies (in which adiposity and telomere length were measured concurrently) and longitudinal studies (in which adiposity was measured in the neonatal period/childhood and telomere length was measured later in childhood or adulthood):**

****

**Although the authors performed two separate meta-analyzes, it would be convenient to explain in Methods section that two analyzes with different specific objectives will be carried out, because I believe that the interpretation of the results of one or another study may not be the same.**

*Response:* *We agree that the analyses have different interpretations, and we have added a sentence to the methods to clarify that separate analyses were carried out.*

*Methods: “Moreover, cross-sectional and longitudinal studies were also meta-analysed separately, since longitudinal studies may provide information on whether an association between telomere length and birth weight tracks across the life course”.*

*Please also see our response to Minor Comment 2, as follows* *:*

*“* *In addition to combining estimates of adiposity at different ages in separate meta-analyses, we also conducted different analyses for binary and continuous exposures”.*

**For longitudinal studies, the authors do not established an age of telomere length measurement. Therefore, it would be interesting for the reader to have a brief discussion about the aim of this specific study. I believe that the manuscript would be more valuable if data on “shortening” of telomere length is analyzed. Is it possible to add this analysis with the data provided in the manuscripts? Is it possible to request this information from the authors of the longitudinal studies?**

*Response:* *We acknowledge that the decision not to restrict to a particular age at telomere length measurement is a limitation of our study; whilst we would have liked to have explored the effect of age using meta-regression, such an analysis would not have been possible due to the small number of studies. We have also included and edited the following paragraph in our Discussion, which discusses the tracking of telomere lengths over time:*

*Discussion: “…an alternate hypothesis is that telomere length is largely pre-determined at birth,[Factor-Litvak et al., 2016, https://www.ncbi.nlm.nih.gov/pubmed/26969272] and that variable rates of attrition in adulthood would not necessarily be enough to alter an individual’s telomere length percentile ranking.[Aviv et al., 2015, https://www.ncbi.nlm.nih.gov/pubmed/25774608] Whilst this does not negate the possibility that oxidative stress later in life may still contribute to attrition, this group state that early determinants of telomere length may be more important.[Factor-Litvak et al., 2016, https://www.ncbi.nlm.nih.gov/pubmed/26969272] Under this assumption, combining estimates of neonatal adiposity in relation to telomere length ascertained at different ages should not alter results appreciably, as each individual would be placed on a set trajectory, altered little by postnatal exposures.”*

*We agree with the reviewer that studying shortening of telomere lengths over time may still be interesting; whilst this was not stipulated in our original protocol, our search strategy should have captured relevant studies examining change in telomere length. However, upon screening the literature for our current analysis, we only detected one paper studying change in telomere length in relation to childhood anthropometric measures. This paper was in the format of a trial, and thus did not meet our inclusion criteria. We therefore think that more studies would be necessary before useful evidence synthesis could be undertaken.*

**3.     To facilitate the pooling of results, all estimates were standardized:**

****

**Perhaps in order to diminish a possible heterogeneity on datasets, the raw data could have been requested to original authors. Besides, this could have increased the number of included studies.**

*Response:* *Standardising estimates per se would not influence the heterogeneity of the results–we have simply put them on a comparable scale. Analysing data on the raw scale would be inappropriate since a one-unit increase in one study is not always the same as a unit increase in another. We note that standardising estimates across studies so that they are on comparable scales in meta-analyses is common practice.*

*Since many of the studies were published several years ago, individual-level raw data were not available (and even after contacting authors, some of the summary-level data required to standardise estimates were also not available). Restricting analyses to those with individual raw data available would have resulted in us being able to combine fewer studies, which would have been problematic, given that we cannot exclude the possibility of non-inclusion bias in our work.*

*We have edited the Discussion to address this point:*

*Discussion: “* *Where possible, we contacted authors to obtain the necessary information to standardise estimates, permitting them to be included in the meta-analysis. However, many of the source publications were written over 15 years ago, and the original data were not available. The meta-analysis may be subject to non-inclusion bias if the studies included in the meta-analyses are different to those not included”.*

**4.     Potential “Publication bias” assessment is missing in the manuscript.**

*Response: We agree that investigating potential publication bias is important. However, we were unable to test for small-study effects (as one indicator of publication bias) using funnel plots, since we had so few studies in each category (the following is a quote from Sterne et al. 2011, https://www.ncbi.nlm.nih.gov/pubmed/21784880): “As a rule of thumb, tests for funnel plot asymmetry should not be used when there are fewer than 10 studies in the meta-analysis because test power is usually too low to distinguish chance from real asymmetry.”*

*We have now mentioned this as a limitation in the Discussion section:*

*Discussion: “Despite finding no strong evidence of non-inclusion bias, we acknowledge that publication bias remains a possibility, and this is therefore a limitation of our work. We were not able to make meaningful inferences about the likely presence of small-study effects using funnel plots, since there were so few combinable studies in each group [Sterne 2011, BMJ, https://www.ncbi.nlm.nih.gov/pubmed/21784880]”.*

**Minor concerns:**

**1.     In the Discussion section, the authors mention that heterogeneity is observed in the meta-analysis of categorical variables, and that this could be due to the cut-off values to define groups. However, at this point the authors should also mention other potential causes of heterogeneity such as the telomere length detection method, ethnicity, etc. Moreover, most studies performed adjustment for potential confounding variables age and sex, but we cannot exclude unmeasured or residual confounding. It is also possible that perinatal factors were involved. For example, data on Entringer 2013 manuscript was corrected for “Obstetric complications, preg. Specific stress” (Table 2), and although in this dataset the estimate was corrected, we cannot assume that other studies were free of perinatal complications.**

*Response: We thank the reviewer for this helpful comment and have now expanded upon these points in the Discussion section.*

*Discussion: “However, heterogeneity is likely to be, at least in part, due to the differing thresholds used to define adiposity categories (e.g. “percentiles of BMI), as well as other potential sources, such as differing ethnicities between studies, and the methods used to measure telomere length”.*

*“The lack of adjustment for prenatal factors in most studies also makes it difficult to establish whether the associations observed are due to a foetal predisposition to larger or smaller body size, or in utero effects. For example, birth weight may act as a surrogate marker for many maternal sources of in utero adversity,[Tyrrell et al., 2016, https://www.ncbi.nlm.nih.gov/pubmed/26978208] and it may be these mechanisms that are important in determining telomere length. A meta-analysis focussing specifically on these exposures would therefore be of value in the field.“*

**2.     In the Methods section, it is not clearly explained that different meta-analysis will be carried out. I would establish a priori that different studies will be done, and according to this, different types of data will be collected. For example, different measurements of “adiposity” were considered for incorporation into the meta-analysis, including weight and birth weight, but weight or birth weight are not measures of adiposity by themselves. Some studies presented estimates of average telomere length by adiposity exposure groups, for example, in obese vs. non obese, and such studies are eligible indeed.**

*Response: We have now added the following to the Methods section to express a priori that different analyses will be carried out to accommodate the different adiposity measures and different study designs.*

*Methods: “* *In addition to combining estimates of adiposity at different ages in separate meta-analyses, we also conducted different analyses for binary and continuous exposures. Moreover, cross-sectional and longitudinal studies were also meta-analysed separately, since longitudinal studies may provide information on whether an association between telomere length and birth weight tracks across the life course”.*

**3.     Perhaps the authors should add a brief paragraph on the meaning of the measurement of telomere length in peripheral blood or cord, instead of the adipose tissue itself.**

*Response: We have added the following to the Methods explaining the utility of measuring leucocyte telomere length:*

*Methods: “* *Leucocyte telomere length is commonly considered as a proxy for ‘whole-body’ ageing and biochemical stress, as well as being a risk factor for disease in its own right (Epel et al., 2008, https://www.ncbi.nlm.nih.gov/pubmed/20195384).”*

**4.     Obeying the fact that the telomere length varies by age and gender, the paragraph on adjustment for age and sex in Methods section, should be written aside.**

*Response: We are sorry that we are unclear what the reviewer is suggesting here. We are happy to take additional advice from the editor to address this point. As the reviewer states, telomere length varies by age and gender and therefore we considered these important potential confounders (as they are also associated with adiposity).*

**5.     Eligible studies included those with at least one measure of adiposity in the neonatal period or childhood (mean age <19 years). Childhood is the age span ranging from birth to adolescence. Perhaps the authors should reconsider the name of the group or the age range.**

*Response:* *We acknowledge the standard definition of childhood as birth to adolescence, and the reviewers are correct that in our manuscript, we do not include the neonatal period in this definition. We have therefore used the term ‘later childhood/adolescence’ instead of ‘childhood’ in the Abstract and Introduction, and in the Methods, to prevent repetition of the longer term ‘later childhood/adolescence’, we have stated that this is what we mean the first time that we use the term ‘childhood’ (‘Inclusion criteria’ of Methods).*

*Methods: “* *Eligible studies included those with at least one measure of adiposity in the neonatal period or later childhood/adolescence (hereafter used interchangeably with ‘childhood’, defined as after the neonatal period [0-28 days], with mean age <19 years).”*

*We hope this is acceptable to reviewers. We will gladly change it to something different if the reviewers have specific suggestions.*

**6.     Please add in the Tables the n of the groups (binary exposures).**

*Response: We have added these numbers as requested.*

**7.     In Table 2, I was not able to find the meaning of the letters (A to F) in the Analysis column.**

*Response: We thank the reviewers for their comment. These letters were initially annotated on each of the panels of the Figures, corresponding to the six meta-analyses we ran. We have now changed the letters to the title of the analysis in each of the six meta-analyses presented e.g. ‘Change in TL [SD] per 1-SD birth weight’ (Figure 1, first analysis), which are also explained further in the legends of each Figure (1 and 2).*

**8.     In Table 2, in the column "Sex Adjustment", in row 4 (De Zegher 2016) and also in row 16 (Shalev 2014) there is a question mark of uncertain significance.**

*Response: We thank the reviewer for noticing this; we have now corrected it.*

**9.     In Figures 1 and 2 the statistics I2 and the p-values are shown. I assumed that the p-value corresponds to the analysis of heterogeneity, but it is actually not clear.**

*Response: These do indeed correspond to the heterogeneity statistic. We have clarified in the legend of each Figure.*

**10.   In Figures 1 and 2 it would be useful for the reader to find a brief explanation on how to read the Forest plot.**

*Response: We have elaborated further in the legend of Figure 1, and also added this information to Figure 2.*
